# Stem Cell Therapy for Diseases of Livestock Animals: An In-Depth Review

**DOI:** 10.3390/vetsci12010067

**Published:** 2025-01-17

**Authors:** Raghavendra B. Narasimha, Singireddy Shreya, Vijay Anand Jayabal, Vikas Yadav, Prasana Kumar Rath, Bidyut Prava Mishra, Sudhakar Kancharla, Prachetha Kolli, Gowtham Mandadapu, Sudarshan Kumar, Ashok Kumar Mohanty, Manoj Kumar Jena

**Affiliations:** 1 Department of Biotechnology, School of Bioengineering and Biosciences, Lovely Professional University, Phagwara 144411, Punjab, India; raghavendra.12114873@lpu.in (R.B.N.); singireddy.12111943@lpu.in (S.S.); 2 Department of Animal Biotechnology, Madras Veterinary College, Tamil Nadu Veterinary and Animal Sciences University, Chennai 600051, Tamil Nadu, India; vijayanand.j@tanuvas.ac.in; 3 Department of Clinical Sciences, Clinical Research Centre, Skåne University Hospital, Lund University, SE 20213 Malmö, Sweden; 4 College of Veterinary Science and AH, Odisha University of Agriculture and Technology, Bhubaneswar 751003, Odisha, India; pkrath@ouat.ac.in (P.K.R.); bidyutpravam@ouat.ac.in (B.P.M.); 5 Devansh Lab Werks, 234 Aquarius Drive, Homewood, AL 35209, USA; sudhakar@devlabwerks.com (S.K.); gowtham@devlabwerks.com (G.M.); 6 Microgen Health Inc., 14225 Sullyfield Cir Suite E, Chantilly, VA 20151, USA; prachetha@microgenhealth.com; 7 Cell, Molecular and Proteomics Lab, Animal Biotechnology Centre, ICAR-National Dairy Research Institute (ICAR-NDRI), Karnal 132001, Haryana, India; sudarshan.kumar@icar.gov.in; 8 ICAR-Central Institute for Research on Cattle (ICAR-CIRC), Meerut 250001, Uttar Pradesh, India; ashok.mohanty@icar.gov.in

**Keywords:** stem cell therapy, mesenchymal stem cells, disease models, pluripotency, livestock

## Abstract

Stem cells are unique, undifferentiated cells that have the ability to both replicate themselves and develop into specialized cell types. This dual capability makes them valuable in the development of regenerative medicine. Current development in stem cell research has widened their application in cell therapy, drug discovery, reproductive cloning in animals, and cell models for various diseases. Although there are substantial studies revealing the treatment of human degenerative diseases using stem cells, this is yet to be explored in livestock animals. Many diseases in livestock species such as mastitis, laminitis, neuromuscular disorders, autoimmune diseases, and some debilitating diseases are not covered completely by the existing drugs and treatment can be improved by using different types of stem cells like embryonic stem cells, adult stem cells, and induced pluripotent stem cells. This review mainly focuses on the use of stem cells for disease treatment in livestock animals. In addition to the diseases mentioned, the potential of stem cells can be helpful in wound healing, skin disease therapy, and treatment of some genetic disorders. This article explores the potential of stem cells from various sources in the therapy of livestock diseases and also their role in the conservation of endangered species as well as disease model preparation. Moreover, the future perspectives and challenges associated with the application of stem cells in livestock are discussed. Overall, the transformative impact of stem cell research on the livestock sector is comprehensively studied which will help researchers to design future research work on stem cells related to livestock diseases.

## Simple Summary

Stem cell therapy in livestock animals is in its infancy, although substantial progress has been achieved in humans. Stem cells are unique cells in terms of their ability to divide and maintain their own population, and to differentiate into many types of cells. The totipotent, pluripotent, multipotent, and unipotent stem cells have different properties and differentiation abilities. The induced pluripotent stem (iPS) cells are man-made stem cells, which have been developed from the differentiated cells of animals or humans by introducing pluripotent genes through a vector system. The present review discusses in detail the current status of stem cell therapy in livestock animals and opportunities for future progress, challenges, and ethical considerations. Mesenchymal stem cells (MSCs) are the adult stem cells with great potential for the treatment of various livestock diseases like laminitis, chronic osteoarthritis, mastitis, endometriosis, recurrent uveitis, etc. The MSCs have immunomodulatory roles and are found suitable in regenerative therapy in livestock through homing and through differentiation in the affected tissue area. This review article provides researchers with comprehensive information on stem cell therapy in livestock and will help them to create strategies for further research in this aspect.

## 1. Introduction

The word “stem cell” was first used to describe the cells that give rise to the germline; Valentin Haecker at the University of Freiburg and Theodor Boveri at the University of Munich both used the word “stem cell” in a similar way [1]. Embryonic stem cells are basically pluripotent in nature, meaning they give rise to the three layers of the germline but are not totipotent, as they cannot give rise to the extra-embryonic cells called the trophectoderm [2]. In the year 1981, embryonic stem cells were first identified, isolated, and cultured in mice by Martin Evans and Matt Kaufmann [3]. Thomson et al. (1998) successfully isolated stem cells from human blastocysts for the first time [4]. The self-renewal property of stem cells is being used to design regenerative medicine for various clinical applications. Embryonic stem cells are found at the blastocyst stage of the developing embryo [5]. Peripheral blood, umbilical cord blood, adipose tissue, bone marrow, blood vessels, brain, skeletal muscles, and liver are the sources of adult stem cells [6]. The bone marrow and umbilical cord blood give rise to two types of stem cells, namely, hematopoietic stem cells (HSCs) and non-hematopoietic or mesenchymal stem cells (nHSCs/MSCs). The HSCs differentiate to form cells of the immune system, blood cells, and platelets. The MSCs have the ability to develop into various cell types like bone, cartilage, ligaments, skin, muscle, and connective tissue. Stem cells can be used to treat livestock diseases, where organs with limited regenerative capacity, such as tendons, ligaments, and cartilages, are affected extensively. Stem cells can modulate the immune system, making them efficient for the treatment of inflammatory and autoimmune conditions in livestock [7]. The key areas of stem cell therapy in livestock include mastitis, laminitis, wound healing, orthopedic conditions, and neurological disorders. Standardization of protocol for stem cell preservation, isolation, and culture is essential for their stable use as therapeutics in many degenerative diseases. The long-term effects of stem cell therapy are essential for clinical applications, and stem cell therapy must be accessible and economical for livestock owners [8]. This article comprehensively reviews the use of stem cells in the treatment of different diseases in livestock animals and discusses the mechanisms by which stem cells heal the diseases, such as the localization of stem cells during transplantation and the molecular interaction.

## 2. Properties of Stem Cells

The properties of self-renewal and differentiation into specialized cell types make the use of stem cells highly valuable in regenerative medicine. They also offer potential therapeutic applications for various diseases in animals and humans [9]. The properties of stem cells which differentiate them from normal cells are described as follows:

### 2.1. Self-Renewal Property

Stem cells have the remarkable ability to replicate themselves, creating identical copies through cell division which maintains a population of undifferentiated stem cells. This self-renewal capacity can last throughout the lifetime of an organism. However, this self-renewal property is questioned sometimes as some stem cells have a finite culture life [10].

### 2.2. Differentiation

Stem cells can differentiate into more specialized cells with specific functions. This ability to differentiate into diverse cell lineages is known as pluripotency or multipotency. The differentiation potential of stem cells can vary based on the type of stem cell [11].

### 2.3. Homing

Stem cells have the ability to migrate to the site of injury or inflammation to carry out their repair mechanisms, which is called homing [12]. Stem cell homing is a multi-step endogenous physiological process, and this mechanism is also used by the exogenously introduced stem cells for tissue regeneration and repair [13].

## 3. Classification of Stem Cells

Based on the origin or source of production and the differentiation ability, stem cells have been categorized into various types, which are discussed below.

(a) Based on origin

Based on the origin of the stem cells, three types exist.

# Embryonic stem cells:

These cells are inherently pluripotent and originate from the inner cell mass of the blastocyst. They have the capability to differentiate into a variety of cell types, leading to the formation of organs and tissues [14]. However, their use and isolation raise ethical issues and pose a risk of immune rejection in many cases [15].

# Adult stem cells:
  These cells are adult-derived stem cells, which are multipotent in nature and are derived from various body tissues and organs. These are mainly used in regenerative medicine due to their availability from multiple sources. The main sources of these cells are umbilical cord blood, dental pulp, adipose tissue, bone marrow, and other organs. They help overcome the risks of immune rejection when the cells are isolated from a patient and are used for the treatment of the same patient [16,17]. The adult stem cells can be further classified into two sub-types:
  - ***Hematopoietic stem cells:*** These are found in abundant quantities in bone marrow and can differentiate into various immune cells, including leukocytes, erythrocytes, and platelets [18].
  - ***Non-hematopoietic stem cells:*** Commonly known as mesenchymal stem cells, these cells have a strong immunomodulatory response. These cells from different organs of the body can develop into distinct cell types such as connective tissue, bone, cartilage, muscle, and fat [19].
# Induced pluripotent stem (iPS) cells:

These are reprogrammed adult somatic cells which have been produced by the transfection of pluripotent genes into the differentiated cells. After a few days of culture, the transfected cells are converted to stem-cell-like cells. This technique was pioneered by Takahashi and Yamanaka in 2006 using mouse somatic cells. These cells do not raise any ethical concerns or pose immune rejection risk, as they are prepared from the patient’s own cells [20]. The challenges in using these cells for treatment include their safety and efficacy, and ongoing research is focused in these aspects [21].

(b) Based on Differentiation Ability

The ability of stem cells to differentiate into different types of cells allows them to be categorized into five main categories.

# Totipotent stem cells:

These cells exhibit the greatest potency, capable of differentiating into any cell type. The cell in the zygote has the potential to develop into a complete organism, and it is totipotent in nature. Totipotent stem cells in animals have the ability to develop into the three primary germ layers (ectoderm, mesoderm, and endoderm) of the early embryo and the extra-embryonic tissue such as the placenta. The mechanism of transition from totipotency to pluripotency is still not clear. Plant cells also exhibit this property [22,23].

# Pluripotent stem cells:

These cells have the ability to develop into the three primary germ layers of the embryo, but have no role in placenta development. These germ layers give rise to the organs and tissues of the body. The embryonic stem cells in the inner cell mass of the blastocyst-stage embryo in animals are the pluripotent stem cells [24].

# Multipotent stem cells:

These cells have a limited range of differentiation ability. Multipotent stem cells can develop into multiple specialized cell types present in a specific tissue or organ. Adult stem cells are multipotent in nature and can be found in various organs and tissues in the body [25]. Some of these cells include adipose-derived stem cells, bone-marrow-derived stem cells, mesenchymal stem cells, and umbilical-cord-derived stem cells [26].

# Oligopotent stem cells:

These cells differentiate into a few cell types in a specific lineage. The myeloid or lymphoid stem cells which give rise to blood cells are the best examples of oligopotent stem cells [27].

# Unipotent stem cells:

These cells can only differentiate into a single cell type. For instance, skin stem cells, which can only produce more skin cells, are unipotent in nature [28].

## 4. Application of Stem Cells in Animal Treatment

The properties of stem cells have enabled their use in the treatment of diseases in animals. Researchers are currently exploring methods of autologous and allogenic treatment for animal diseases. There have been many cases of stem cell use in animal treatment and the therapeutic use of stem cells has been found to be safe and beneficial for animal health [29]. Adipose-derived MSCs have been used to treat chronic osteoarthritis, while umbilical-cord-blood-derived MSCs have shown positive results in many degenerative diseases without any adverse effects. The applications of stem cells are being explored for treating inflammatory diseases in animals and developing drugs for humans using animal models [30]. Further study is needed in the areas of stem cell isolation, in vitro propagation, storage, and characterization, for use in different diseases [31].

The treatments of osteoarthritis and bone spavin with MSCs and adipose-derived stem cells, respectively, have shown convincing results [32]. Studies on the development of stem cell therapy for bovines have revealed goats as a suitable model. Goats are being used to study autologous stem cell therapy for cartilage regeneration [33]. Scrapie, a neurodegenerative disease in sheep, is now being treated with the help of bone-marrow-derived and peripheral-blood-derived stem cells and is under investigation for the development of regenerative medicine [34]. Tissue regeneration has been observed in cartilage and bone tissues using MSCs, in addition to their use in many disorders like central and peripheral nervous system disorders; gastrointestinal system disorders; and respiratory, reproductive, urinary, integumentary, and endocrine disorders including eye and adnexa ailments [35].

For MSCs to be used in cartilage regeneration, they require support from the microenvironment for the differentiation process. To provide the required microenvironment, various growth factors and chemicals have been used including transforming growth factor (TGF)-beta, bone morphogenetic protein (BMP), fibroblast growth factor (FGF), dexamethasone, ascorbic acid, and transferrin [36]. The in vitro culture system has supported the growth and differentiation of MSCs by the use of scaffolds and matrices to mimic the extracellular matrix (ECM) and enhance the MSC chondrogenesis [37]. Studies conducted on animals like sheep, goats, and horses have revealed that the quality of cartilage regeneration varies among animals, and it does not fully replicate the natural cartilage. It has shown promising results with improved defect fillings and fibrocartilage formation. Studies revealed positive results in the treatment of osteoarthritis in horses and dogs, with reduced pain and lameness and reduced disease progression [36].

The capacity of the MSCs to differentiate into osteoblasts (bone cells) has promoted their use in bone regeneration and bone tissue engineering applications. Researchers have explored a case for treating pastern joint arthrodesis in horses using stem cells [38]. In this study, a section of bone was surgically removed, and bone-marrow-derived MSCs, along with scaffold and matrix materials, were supplemented. This approach enhanced bone formation and resulted in successful arthrodesis compared to the control group, which only received compression plates [39]. While the outcomes have been positive and significant in bone regeneration, challenges remain regarding the standardization of the isolation, culture techniques, and transplantation of the stem cells, as well as understanding the mechanism of action of the MSCs and the long-term effects of these cells in treating arthritis and bone fractures [40].

Wobbler syndrome, also known as cervical vertebral malformation (CVM), is a condition that impacts an animal’s musculoskeletal and neurological systems, mainly occurring in dogs and horses. The clinical signs include spasticity, ataxia, and lack of coordination due to narrowing of the spinal canal [41]. The studies using MSCs for the treatment of wobbler syndrome have revealed that bone-marrow-derived MSC administration to the intrathecal region is safe. However, the neurological function of the horse did not seem to improve [42]. In another study, adipose-derived MSCs were administered to the atlanto-occipital regions in horses with a common form of wobbler syndrome. This study concluded that the atlanto-occipital injection is more effective for cell distribution towards the affected regions in the spinal canal [43]. Further research is needed to improve the efficacy of the studies and the mechanism of action, and to develop comparative studies between stem cell therapy and chemically derived drug therapy [44].

Mastitis in cattle is mainly caused due to injuries and infections. The use of antibiotics for the treatment of mastitis has led to the issue of antibiotic resistance in the disease treatment. To address this, a new treatment strategy has been developed using stem cells. The MSCs are injected with a gene resistant to microbes like *Staphylococcus aureus* and *Escherichia coli*. These stem cells are then injected into the mammary glands, where they replace the infected cells and replicate, forming healthy tissue in the affected area. This method has shown a lower number of somatic cells in the milk. The MSCs secrete antimicrobial peptides that inhibit the further development of the infection by creating an anti-inflammatory environment and disrupting the integrity of the microbe’s cell wall. The research was performed on a total of 39 cows, 36 of which had mastitis. This resulted in the identification of the concept that bone-marrow-derived stem cells are more effective in treating mastitis caused by *S. aureus* and the adipose-derived stem cells have more significance in treating the infections caused by *Enterobacteriaceae* and *E. coli* [45]. The main reason for developing an alternative treatment for mastitis was the reduced milk production in successive lactations in infected cattle due to extensive mammary epithelial cell damage in the mammary gland. The milk with a high somatic cell count led to decreased shelf life and a change in chemical composition. This was the main reason for reducing the use of antibiotics, as they resulted in elevated somatic cell counts in milk [46].

## 5. Immunomodulatory Role of MSCs Through Paracrine Signaling

The MSCs are being extensively studied for therapeutic applications in veterinary science due to their ability to differentiate into various cell types and their immunomodulatory properties. While MSCs’ differentiation potential is important for tissue regeneration, their paracrine functions, particularly in inflammation regulation, are also key to their therapeutic effects. MSCs can modulate the immune response by interacting with immune cells and secreting factors that suppress inflammation. They primarily act through paracrine mechanisms by secreting molecules that affect neighboring cells, promoting tissue regeneration and repair [47]. Cytokines, growth factors, and extracellular vesicles, which constitute the secretome of MSCs, contribute to their regenerative and therapeutic effects by regulating inflammation, promoting angiogenesis, and supporting tissue repair. MSCs secrete some factors such as bFGF, TGF-β1, and βNGF which have specific functions in signaling and immunomodulation. It is seen that MSCs exhibit low immunogenicity due to the low expression of major histocompatibility (MHC) class II molecules and co-stimulatory molecules [48]. They are activated by inflammatory cytokines such as interferon-γ (IFN-γ) and tumor necrosis factor-α (TNF-α) to enhance immunomodulatory activities. Upon activation, MSCs release immunosuppressive factors including prostaglandin E2 (PGE2), which suppresses T-cell proliferation, and indoleamine 2,3-dioxygenase (IDO), which inhibits T- and B-cell proliferation [49].

MSCs exhibit anti-inflammatory properties through paracrine mechanisms, where they secrete a variety of bioactive molecules that modulate the immune response and promote tissue repair. MSCs can induce the polarization of macrophages from a pro-inflammatory (M1) phenotype to an anti-inflammatory (M2) phenotype, which promotes tissue repair [50]. MSCs can decrease the production of reactive oxygen species (ROS) through neutrophils. The MSC-derived exosomes, which are nano-sized vesicles containing various bioactive molecules, deliver MSC-sourced factors directly into immune cells and injured tissue, contributing to the anti-inflammatory effects. Due to these properties, MSCs have shown promising results in treating immune-mediated diseases, dermatological issues, and other conditions. The MSCs have been studied for their use in treating atopic dermatitis, furunculosis, anal vasculitis, and scar tissue regeneration [51].

## 6. Development of Disease Models

Disease models are important in drug discovery and research involving the molecular pathophysiology of various diseases. To develop a disease model, stem cells are first taken from a particular animal, and diseased cells are injected into those stem cells in vitro. These modified stem cells are then injected back into the same animal, which is now considered as a disease model [52]. The crucial role of stem cells in veterinary regenerative medicine and clinical practice has been reviewed by El-Husseiny et al. (2022) and Markoski (2016) [53,54]. Currently, stem cell therapy is available for many diseases affecting livestock animals. A few of them are described below:

### 6.1. Laminitis

Laminitis is a debilitating disease that affects the feet of horses and other ungulates. It is characterized by damage to the sensitive laminae that connect the pedal bone to the inner hoof wall, which can lead to the pedal bone sinking and rotating within the hoof capsule. The pathogenesis of laminitis involves several factors that disrupt the normal function of the laminae including inflammation, endocrinopathy, and trauma [55]. One of the studies used adipose-derived mesenchymal stem cells (aMSCs) to administer locally, along with platelet-rich plasma (PRP), to the horses with chronic laminitis. This combination was used on a group of nine horses which had shown unsatisfactory outcomes to the previous treatments. Based on the collected results, it was observed that the aMSCs employed in the study promoted the healing of laminitis and expressed some markers including CD90, CD105, CD44, and CD73, but not others like CD45, CD31, and CD34. The aMSCs express markers linked to the biological response to tissue injury including IL1-Ra, STC-1, TGS-6, and SDF-1. In horses with flexor tendinitis, the use of PRP in conjunction with allogenic aMSCs was safe [56]. Godwin et al. (2012) used mesenchymal stem cells for the treatment of tendon injuries in race horses and observed the long-term efficacy of MSCs in tendinopathy treatment. In this study, 141 race horses were studied and followed up for 2 years after the therapy. The reinjury percentage of the race horses (*n* = 113) was 27.4% in the follow-up period of 2 years, which showed better results as compared to previous studies, and this strategy can be translated to human tendon injuries [57].

### 6.2. Equine Recurrent Uveitis (ERU)

This is an immune-mediated condition that can cause blindness and is characterized by recurrent or chronic inflammatory episodes. A study revealed that CD4^+^ T-cells in horses with ERU have an active phenotype. This may indicate an increased level of immune system activation, which is a feature of autoimmune diseases. Activated T-cells suggest a continuing inflammatory response that is involved in the pathophysiology of ERU disease [58]. The study investigated MSCs’ capacity to alter the immune system. MSCs are well known for their capacity to affect T-cell behavior by immunosuppressive action. The researchers co-cultured MSCs with CD4^+^ T-cells from horses affected by ERU, in the in vitro experiments. The results demonstrated that MSCs could alter the activation status of CD4^+^ T-cells, suggesting a therapeutic potential for MSCs in treating ERU by moderating the overactive immunological response [59].

### 6.3. Endometriosis

Endometriosis is a condition that can lead to infertility. A study conducted to evaluate the histological and immunohistochemical changes in the endometrium by using endometriotic biopsies revealed morphological alterations, such as the presence of fibrotic stromal cells and altered glandular epithelia in mares [60]. The study emphasized that the endometrial tissues contain vimentin and laminin. Smooth muscle alpha-actin (SMA) expression was assessed both prior to and following MSC transplantation. Day 0 saw the presence of SMA in periglandular fibroblasts and uterine glands; however, day 7 showed no evidence of SMA expression, indicating a decrease in myofibroblast activity following therapy. The alterations in protein expression and the histological features seen in the biopsies suggested that the infusion of equine adipose-derived mesenchymal stem cells (eA-MSCs) may have encouraged a regenerative response in the endometrium. According to the study, MSC therapy may aid in the restoration of the endometrial architecture and function, which could enhance the prognosis for fertility in mares with endometriosis [61].

### 6.4. Persistent Breeding-Induced Endometritis (PBIE)

This condition is a significant cause of infertility in equines, characterized by an exaggerated inflammatory response to spermatozoa. A study utilized mares that were experimentally induced with PBIE. Mares received either endometrial MSCs, adipose MSCs, or a control treatment. The inflammatory markers, clinical signs of endometritis, and the engraftment of MSCs in the uterine tissue were monitored over a specified time period [62]. Both types of MSCs demonstrated significant anti-inflammatory properties, effectively reducing the inflammatory response in the uterus when compared to the control group. This was evidenced by lower levels of pro-inflammatory cytokines and a reduction in neutrophil infiltration. The study assessed the engraftment of MSCs, capable of migrating to and integrating within the uterine environment, contributing to tissue repair and regeneration. While both MSC types showed beneficial effects, there were differences in their efficacy, with endometrial MSCs potentially offering greater advantages in modulating the inflammatory response [63].

### 6.5. Equine Metabolic Syndrome (EMS)

A case study that aimed to use autologous adipose-derived mesenchymal stem cells (ASCs) treated with 5-azacytidine (AZA) and resveratrol (RES) improved metabolic liver function in a horse that had equine metabolic syndrome (EMS). This horse, a nine-year-old Dutch warmblood, was diagnosed with severe obesity and insulin resistance, which are typical in EMS. The horse was treated with three intravenous injections of AZA/RES-treated ASCs at weekly intervals. The therapeutic effect was assessed by measuring liver-specific enzymes in the blood. The study found that ASA transplantation reduced the levels of glutamate dehydrogenase (GLDH), gamma-glutamyl transferase (GGT), lactate dehydrogenase (LDH), and aspartate transaminase (AST). These enzymes are typically elevated in horses with EMS, suggesting liver impairment. It was concluded in the study that autologous, rejuvenated ASC injections can be a beneficial therapeutic intervention for EMS and can improve liver metabolism. The study also indicates that MSCs initially travel to the lungs after injection and then to the liver, which makes them useful in treating disease that are affecting liver function [64].

### 6.6. Cutaneous Wound

A pilot study was conducted to assess the use of intravenously administered allogeneic cord-blood-derived multipotent mesenchymal stromal cells (CB-MSCs) to promote the healing of surgically created cutaneous wounds in horses [65]. Two horses were given fluorescently labeled CB-MSCs after the creation of wounds on their forelimbs and thoraxes. The objectives of this study were to determine the safe dose of intravenously administered CB-MSCs, and to observe the pattern of homing and engraftment in the wound biopsies. The results indicated that the horses did not develop any adverse effects on the infusion of CB-MSCs and all vital signs remained normal. The study also found that preferential homing and engraftment of the CB-MSCs to the wounds occurred with the cells persisting at the wound sites up to 33 days after the wound creation. The homing of CB-MSCs to the wound sites was observed as early as on day 1, and by day 33, cell-like structures consistent with the CB-MSCs appeared to be integrated into the interstitium. This study suggests that intravenously administered CB-MSCs are safe and can effectively home and engraft to cutaneous wounds in horses. These findings are crucial for further research in developing regenerative medicine for horses [65]. A study with the use of heterologous Wharton’s jelly-derived MSCs on the large chronic skin wound of a six-month-old filly revealed promising results [66]. In the beginning, the mean wound area was 7.28 ± 0.2 cm^2^. Four days after the use of MSCs, the wound area was reduced to 1.90 ± 0.03 cm^2^. The mean wound area was 0.38 ± 0.01 cm^2^ during discharge, with a healing rate of 80%, and after another 5 days, the wound was completely healed.

### 6.7. Osteoarthritis

Evaluation of the effect of the intra-articular injection of autologous adipose-derived MSCs in osteoarthritic dogs revealed that the limb functions of the dogs were improved [67]. In this study, 10 lame dogs with osteoarthritis were treated with the MSCs and the results were analyzed by measuring the peak vertical force (PVF) and vertical impulse (VI) in the treated dogs. The results showed significant improvement in the mean value of PVF and VI within the first 3 months of the treatment with increases of 9% and 2.5% body weight, respectively, at day 30. However, the effect of adipose-derived MSCs lasted for 3 months post-treatment and subsequently reduced, showing its transient effect on the healing of osteoarthritis [67].

## 7. Introduction to iPS Cells

Induced pluripotent stem (iPS) cells are reprogrammed adult somatic cells which are prepared by reprogramming the differentiated cells into stem-cell-like cells using a set of pluripotent genes through transfection and gene expression. They were first developed by Takahashi and Yamanaka using mouse somatic cells in the year 2006, and later in 2007 using human somatic cells [68,69]. They are generated by introducing a specific set of genes, called pluripotency transcription factors, which reprogram the somatic cells to acquire the properties of pluripotent stem cells. The transcription factors inserted into the somatic cells are the same as those found in the embryonic stem cells which include OCT4, SOX2, NANOG, c-Myc, and KLF4 [70]. These transcription factors play specific functions that enable the cells to become pluripotent. Disease modeling, medication development, and regenerative medicine are a few applications of iPSCs in veterinary science, besides their wide applicability in humans. They are preferred due to the absence of any ethical concerns or immune rejection, as the cells are derived from the patient’s own body [71]. In total, 24 different genes can be used for reprogramming adult somatic cells into iPSCs [72]. Some of the main transcription factors (pluripotent genes) and their specific functions are mentioned below in Table 1.

## 8. Applications of Induced Pluripotent Stem Cells

### 8.1. Disease Modeling

As the iPSCs are directly derived from the patient’s cells, they can accurately reflect that patient’s genetic makeup, making them highly useful for disease modeling. This is particularly important for studying inherited diseases and genetic disorders. The iPSCs allow for the development of precision medicine specific to the patient’s genetic makeup. This is advantageous over the other types of stem cells as they do not contain the genetic complexity of a particular disease. These models can further be used to test gene therapies and study disease mechanisms [83].

### 8.2. Unlimited Stem Cell Source

The iPSCs mimic the properties of embryonic stem cells, and thus they can be used as a source of stem cells. This ease of access is due to the standardized protocols for their isolation, propagation, and storage [84]. The availability of iPSCs makes them suitable for large-scale drug screening. These cells can also be used to develop particular cell lines using the patient’s somatic cells for the study and for drug designing against a disease [85].

### 8.3. Recapitulation of Human Development

As these cells are stem-cell-like cells, iPSCs mimic the natural development of cells into organs or tissues, as happens in the body. This allows researchers to study the stages and mechanisms of the disease in various cell types specific to a patient. Consequently, iPSCs enable the development of precision medicine by revealing the disease mechanism, leading to the development of safer and effective treatment methods specific to the genetic makeup and disease profile [86].

### 8.4. Ophthalmology

The iPSCs have the ability to differentiate into photoreceptor cells and retinal pigment epithelium cells when exposed to certain factors [87]. These cells can be used to treat age-related macular degeneration. By replacing damaged retinal cells with iPSC-derived cells, there is potential for restoring vision and regenerating the retina. In pre-clinical studies involving transplantation onto a knockout rat, the transplantation neither improved nor worsened the patient’s vision. This study concluded that the use of iPSCs may be a safe and viable treatment for macular degeneration disease [88].

There are limited studies in livestock animals where iPSCs have been used as therapeutics. Some of them are discussed here.

### 8.5. Inherited Skin Diseases

Epidermolysis bullosa (EB), a genetic skin disorder commonly found in calves, buffalo lambs, and foals is caused by a missense mutation in the COL7A1 gene, which encodes type VII collagen. This is characterized by severe blistering and scarring of the skin. There is no cure for this disorder and treatment options are limited to managing the symptoms and disease progression. This is where iPSCs offer a promising therapeutic approach for EB. This is accomplished by generating patient-specific skin cells that are identical and carry the mutation. These cells can further be edited using advanced gene-editing techniques to correct the genetic defect in the derived iPSCs. Once corrected, these iPSCs can be differentiated into healthy keratinocytes or other skin cells suitable for transplantation. The differentiated cells can be transferred autologously to the patient, helping regenerate damaged skin and improving the symptoms of EB. This technique has potential applications for treating EB in humans as well [89].

### 8.6. Liver Failure

The iPSCs are also used to develop organoids that mimic the complex structure and function of organs in the body. When a patient’s liver is damaged, it often requires transplantation, which depends on finding a suitable donor. In a study, it was seen that the administration of adipose-derived mesenchymal stem cells intravenously, to treat experimentally induced acute hepatic damage, was successful in decreasing the liver enzyme levels in the blood and restored the liver structure in canines. This application can be explored more in other livestock animals for the treatment of liver-related diseases [90].

### 8.7. Pig as a Model for iPS Cell Study

Xu et al. (2019) successfully developed pig iPS cells from pericytes and embryonic fibroblasts, using a retroviral vector system encoding Oct4, Sox2, Klf4, and cMyc genes [91]. The iPS cells were cultured in modified LCDM (consisting of hLIF, CHIR99021, (S)- (+)- dimethindene maleate, and minocycline hydrochloride) medium and stable cells were produced which were characterized by the expression analysis of pluripotency markers [91]. Yang et al. (2013) successfully generated pig iPS cells from fibroblast cells which expressed the pluripotency markers [92]. Furthermore, these pig iPS cells were differentiated to form neural cells and it was found that these cells could be differentiated to form neurons, astrocytes, and oligodendrocytes of the central nervous system. This study indicated that pigs can be a useful model to study autologous neural iPSC therapies in a system similar to humans.

## 9. Mechanism Involved in the Differentiation of Stem Cells

The surrounding microenvironment affects how stem cells differentiate, providing specific signals that guide them to develop into particular cell types. During tissue repair, stem cells interact with neighboring cells via paracrine signaling, promoting these cells to differentiate into mature cell types [93]. Stem cells also release bioactive molecules, collectively known as secretome, to direct other stem cells to the site of injury, where they aid in tissue regeneration by differentiating into that specific cell lineage. The secretome includes growth factors, cytokines, chemokines, extracellular vesicles, and immunomodulatory molecules [94]. All these molecules have different functions in the paracrine signaling of the stem cells. These molecules will act on the surrounding cells and progenitor cells to promote tissue regeneration [95].

**Figure 1:**
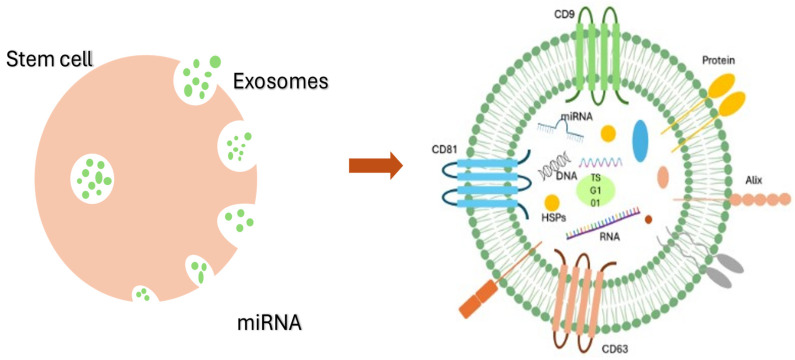
Schematic diagram of stem cells and exosomes. The stem cells expressing CD9 and CD81 mediate further molecular signaling to promote the migration and differentiation of stem cells at the tissue injury site.

Once the signaling molecules enter the neighboring cells, they activate the heat shock proteins, which play a role in the folding and re-folding of native and other essential proteins facilitating the recovery of damaged cells [96]. For example, if the skin is damaged, the platelet accumulation promotes the hemostatic role where the stem cells induce the immune response and produce an anti-inflammatory effect to prevent the skin from further damage. The stem cells then promote the proliferation and differentiation of fibroblasts which replace the damaged skin and inhibit the scar formation which can be seen in most of the wound-healing cases. The CD9 and CD81 are the surface markers of the exosomes which mediate intercellular communication. These surface markers modify adhesion, migration, proliferation, and survival of the cells [97]. The depiction of the stem cells secreting the exosomes and the schematic structure of the exosomes are given in Figure 1:

The CD receptors, which are part of the tetraspanin family, can be used to detect the extracellular vesicles released by stem cells [98]. The HSPs in exosomes are mostly known as circulating markers, which mediate intercellular communications with the injury microenvironment; these can also be used to develop cancer vaccines [99]. The alix and TSG101 proteins are required for the transport of exosomes. Overexpression of TSG101 will increase the secretion of exosomes. Alix, on the other hand, will increase the therapeutic effects of the exosomes. The interaction of alix with ESCRT helps in exosome formation [100,101]. The miRNA helps in regulating cell processes, promoting tissue regeneration, and reprogramming and modulating inflammatory responses [102]. All the molecules expressed on the surface of exosomes and inside the exosomes play crucial roles in stem cell differentiation, intercellular interactions, and tissue regeneration (Figure 1).

Recent research has shifted the traditional understanding of stem cells, which was primarily based on their ability to directly differentiate into specific lineages for tissue repair. Now it is well established that stem cells primarily use paracrine signaling for their differentiation during the tissue repair mechanism [103]. When stem cells are transplanted into the body, they have a very low survival rate. This is compensated for with paracrine signaling where the transplanted stem cells secrete various signal molecules [104].

For instance, if a veterinarian treats a cow with liver failure with the use of stem cells, the stem cells are isolated from the bone marrow of the cow itself; these cells, when administered, are attracted by signaling molecules secreted by the damaged tissue. This occurs by a process of the chemoattractant gradient. Stem cells have receptors, CXCR4 and integrin-β1, which bind to these signal molecules. This process of traveling is called homing [105]. After reaching the site of injury, the stem cells release a set of molecules which helps them to engraft to the damaged tissue leading to stem cell differentiation. The molecules released by the stem cells are growth factors, anti-fibrotic factors, pro-angiogenic factors, and cytokines [106]. The stem cells also secrete exosomes which enhance the therapeutic effects by creating an immunomodulatory environment contributing to the healing and restoration of the damaged liver tissue. Monitoring and follow-up should be performed to report any adverse effects and the extent of healing by stem cells [107].

The signaling molecules involved in paracrine signaling are given in Table 2.

## 10. Challenges in Using Stem Cells for Livestock Diseases

Obtaining enough stem cells is challenging as adult stem cells are limited in tissues. The adult stem cells for the stem cell therapies primarily used in veterinary practices are hematopoietic and non-hematopoietic adult stem cells. For instance, there is only one cardiac stem cell for every one thousand cardiomyocytes, which makes it very limited for the isolation of cardiac stem cells. Adult stem cells also vary with varying physiology [122,123]. In addition, molecular characterization of the adult stem cells is not widely studied [124]. Protocols for isolating and characterizing the stem cells from different tissues or organs are not standardized so far. Adult stem cells have limited proliferation with extensive passaging which makes it difficult to culture them for a long time to enhance their number [125,126]. Contamination from various sources, including that from fetal bovine serum in mesenchymal stem cell culture, creates challenges for their in vitro maintenance [127]. The clinical use of stem cells is limited by their dynamic complexity, biology, potential for teratoma formation, and histocompatibility [128]. A series of regulations must be adhered to in order to address the ethical issues raised by the use of stem cells, especially embryonic stem cells. The use of allogenic stem cells comes with a challenge of immune rejection by the recipient animal, which can be overcome by the use of iPSCs. As the numbers of stem cells obtained or isolated are very limited, the scaling up of these stem cells into large and pure batches for therapeutic uses is challenging [71]. There is still extensive research being undertaken to understand the underlying mechanisms of stem cells, which are yet to be understood completely. The techniques involved in stem cell therapy demand highly equipped and specialized laboratories and personnel, which makes them expensive to establish.

Current stem cell therapies face significant hurdles that limit their clinical application including immune rejection, the potential for teratoma formation, and challenges in cell survival and engraftment. Immune rejection is a major concern where the recipient’s immune system recognizes the transplanted cells as foreign and starts an attack on them. This is due to the presence of major histocompatibility complex (MHC) molecules on the surface of the transplanted cells. Even when using autologous transplantation, there can be issues such as the possibility of adverse effects at the stem cell harvesting area [129]. Moreover, the risk of tumorigenicity is particularly associated with the use of embryonic stem cells and iPSCs as these cells can potentially form tumors. Genetic instability and epigenetic changes in iPSCs during culture can further increase this risk. The limited survival and poor engraftment ability of transplanted cells also present a major limitation, with many cells dying shortly after transplantation due to factors like inflammation and physical stress. In some cases, the cells may fail to integrate properly into the surrounding tissues, limiting their therapeutic effectiveness [130].

The variability in stem cell properties is due to factors like age of donor, health condition, and tissue source. These factors make it difficult to achieve consistent results across different treatments. The lack of standardized protocols for stem cell isolation, characterization, and application further hinders clinical transplantation [131]. Specific to the delivery of stem cells, there are multiple factors that need to be optimized including the route of administration, dosage, and timing. For example, the delivery of stem cells to the brain by intravenous administration can be limited, with some cells accumulating in peripheral organs like the lungs, liver, and spleen. Ethical concerns related to the use of ESCs also pose a challenge. Moreover, challenges remain in identifying a pure population of stem cells, especially adult stem cells (ASCs) in different tissues of various species, and there is a lack of consistent markers among all species [132].

To overcome these limitations, several strategies are being explored. Using autologous cells, derived from a patient’s own body, can eliminate the risk of immune rejection. However, this is not always possible, and in that case, the use of the immunomodulatory properties of MSCs will be helpful. Genetic modification of stem cells can also enhance their survival, engraftment, and therapeutic efficacy [133]. Stem cell preconditioning procedures and improved delivery methods, including intranasal administration and tissue engineering approaches, are being developed to be used in stem cell therapies. The MSC-derived exosomes are being explored to deliver therapeutic factors, which can overcome the risk of immune rejection. Standardizing protocols for stem cell therapy, which includes better methods to characterize and quantify stem cells using better technologies, is crucial for improving the consistency and efficacy of stem cell therapies. Further investigation on the action of stem cells and the use of animal models can lead to better clinical applications [134].

To overcome these challenges, extensive research has to be performed in the field of stem cells for veterinary science. Current research is taking place to identify and develop various stem cell therapies for different diseases with safety and efficacy studies underway [135]. The stem cell therapies have not yet been completely adopted due to the doubt of the safety, efficacy, and cost of these therapies. The use of stem cell therapies can be widely adopted once the research findings reveal their safe and efficient outcome in livestock disease treatment [136].

## 11. Requirement of Standardized Guidelines for Stem Cell Use

Standardized guidelines are crucial for the safe and effective use of stem cells in both research and clinical applications. The intrinsic heterogeneity of stem cell features, together with the complications surrounding their isolation, culture, and applications, mandate strict and uniform protocols, and without such standards, reproducible results cannot be assured. It became a necessity to have clear and strict protocols with respect to the components pertaining to inherent differences within stem cell properties, applied isolation, and culturing methods as well as treatment regimes. Achieving them would ensure the reproducibility of results, treatment safety, or general progress in the field [137]. Standardized guidelines are crucial in minimizing the heterogeneity in stem cell preparations, ensuring the safety of stem cell therapies, facilitating comparisons across different studies, enabling clinical translation and regulatory approval, determining the cell-specific dose of stem cell treatments, and providing parameters that could predict the efficacy of cell-based medicinal products. The absence of standardized guidelines also hinders the comparison of results across different studies. Different research groups may use varying methods for stem cell isolation, proliferation, and applications, making it challenging to evaluate the effectiveness of different treatments. Standardized protocols and assessment criteria would allow for a better comparison of outcomes and facilitate the identification of best practices [138].

## 12. Current Trends of Stem Cells in Veterinary Science

Stem cell therapy is growing in veterinary science with extensive research currently taking place. MSCs have been more preferred for stem cell therapy in livestock diseases. These readily available cells can be separated from a variety of sources including bone marrow, adipose tissue, peripheral blood, and the umbilical cord [128]. There is evidence of the use of MSCs in the treatment of livestock diseases like degenerative diseases, liver failure, and tendon and ligament damages [139]. One of the applications of stem cells is their use for the conservation of endangered animal species [140]. Current research is underway to create a uniform stem cell therapy protocol for a wide group of degenerative diseases. MSCs are found to be promising in stem cell research with easy isolation and expansion in the in vitro conditions. Furthermore, research is being conducted to explore the potential of other types of stem cells in treating degenerative diseases [141].

Numerous uses of stem cells have been identified, including the development of disease models using iPSCs, the use of MSCs for various diseases, the use of bone-marrow-derived stem cells for the treatment of osteoarthritis, etc. Understanding the molecular dynamics of stem cell function will pave the way for the wide acceptance of stem cells as therapeutics for many complicated diseases [142,143].

## 13. Ethical Considerations in Stem Cell Therapy

Significant ethical concerns are brought up by the source of stem cells employed in therapy, especially embryonic stem cells (ESCs). Because using ESCs entails destroying embryos, there is discussion on the ethical standing of embryos and a need to find other sources. There are drawbacks to substitutes like induced pluripotent stem cells (iPSCs) and adult stem cells (ASCs). ASCs may exhibit variable traits and might be challenging to isolate in pure populations. Although iPSCs avoid the moral dilemmas associated with ESCs, questions about possible genetic alterations still exist. Animal welfare is another important ethical factor. Animals may experience pain and discomfort as a result of stem cell treatments, especially those that involve harvesting techniques like bone marrow aspiration. Furthermore, because lower success rates require the use of more animals to accomplish desired results, the efficacy of these procedures is critical for animal welfare [144].

Additionally, there are worries regarding immunological reactions and possible pulmonary problems when using fetal bovine serum in MSC treatments. One important issue that needs to be addressed is the absence of uniform laws controlling stem cell treatments in veterinary medicine. This lack of supervision may result in the use of treatments that have not undergone thorough safety and effectiveness assessment. It is also important to address how the general public views and comprehends stem cell therapies, especially in light of the possible hazards and advantages. Concerns over the instrumentalization and possible exploitation of animals are also raised by the idea of employing genetically modified animals as “bioreactors” to create industrial proteins and human medicinal goods [145].

## 14. The iPSCs and Regulatory Concerns

As a potential remedy for the ethical issues surrounding embryonic stem cells (ESCs), the development of induced pluripotent stem cells (iPSCs) has created new opportunities in regenerative medicine. Reprogrammed from adult somatic cells, induced pluripotent stem cells (iPSCs) can differentiate into any type of cell in the body, offering an almost limitless supply of patient-specific cells for therapeutic uses. Nevertheless, despite iPSCs’ enormous promise, a number of regulatory issues demand careful consideration before they are widely used in clinical settings [146]. Ensuring the safety and dependability of iPSC creation and manipulation is one of the main concerns. Unintentional genetic alterations may be introduced into the patient’s genome by the reprogramming and gene correction techniques used to produce iPSCs, raising the possibility of tumorigenicity. According to the sources, undifferentiated iPSCs have the same propensity to develop into teratomas as ESCs. The iPSC lines must undergo thorough testing and validation in order to reduce the possibility of tumor development and genetic instability [147].

Another regulatory problem is the variability of iPSC lines. Standardizing iPSC-based cell products for therapeutic applications can be challenging due to the variability in iPSC lines, which might affect the consistency and predictability of differentiation into desirable cell types. Addressing this heterogeneity and guaranteeing the caliber and repeatability of iPSC-based treatments requires the establishment of strong selection criteria for iPSC-derived cells, such as cell-specific markers, proliferation rate, lifespan of cells, and genomic analysis [148]. The transition of iPSC-based treatments from the laboratory to the clinic may be hampered by the absence of established regulatory frameworks. To promote the iPSC-based therapy and safeguard patient welfare, thorough rules must be established which particularly address the safety issues, effective adoption, and ethical use of iPSCs [146]. One major obstacle to the broad clinical implementation of iPSC technology is its high cost. The development and delivery of iPSC-based therapies are costly due to the intricate processes involved in iPSC creation, characterization, and differentiation, which call for specific knowledge, equipment, and resources. Intensive research and development initiatives are required to streamline the iPSC production procedures, lower the cost of production, and increase the accessibility of these treatments [131].

## 15. Future Perspectives

With the current research on the use of stem cells for treating various livestock diseases, there are challenges which need to be addressed to have a wide application of stem cell therapy in the livestock sector. One of the key areas of optimization is to determine the appropriate stem cell dosage for the therapy. The stem cell dosage makes it difficult to compare the results across different studies to develop a standardized stem cell dosage for widespread use [149]. Another aspect lies in the understanding of the impact of stem cell processing techniques and cell source on the treatment outcomes. Specific types of stem cells show better results in particular diseases than in all the diseases [149]. One of the main areas of interest of researchers is to develop standardized protocols which include cell dose and administration route. Establishing standard protocols would facilitate the development of guidelines for veterinary stem cell applications [150]. Stem cells not only help in the treatment of diseases but also can help in reproduction, such as in assisting in preserving endangered animal species. The same stem cells can also be used to develop transgenic animals with desired applications [140]. Moreover, the development of iPSC technology can also be explored in developing stem cell therapy for livestock diseases along with their application in developing disease models [151]. The emerging genetic engineering tool CRISPR-Cas9 can be explored for enhancing livestock productivity and disease resistance and for developing new therapeutic strategies with precise gene editing. The stem cell technology can be used to develop more targeted therapy in livestock animals [152].

## 16. Conclusions

Stem cell therapy is rapidly emerging as a promising field in veterinary medicine for treating various diseases in livestock animals. The potential of stem cells lies in their unique properties of self-renewal and differentiation into specialized cell types. Stem cells offer a potential alternative treatment option for diseases that are currently difficult to manage using conventional methods. MSCs have shown promising results in treating various livestock diseases such as mastitis, osteoarthritis, and wobbler syndrome. Studies have demonstrated the efficacy of MSCs in reducing inflammation, promoting tissue regeneration, and improving clinical outcomes in animal models. The iPSC technology developed for reprogramming somatic cells into stem-cell-like cells has immense potential in developing disease models and therapeutic applications. Using this technology, researchers will be able to develop disease-specific cell lines which will enable them to study disease mechanisms and drug discovery, as well as help in the development of tailored stem cell therapies. Despite these encouraging developments, there are still some challenges to popularize stem cell therapy in clinical settings. These include the need for standardized protocols for the isolation, culture, and characterization of stem cells, as well as ensuring their safety, efficacy, and affordability. In addition, the optimization of stem cell dosage and administration routes, the investigation of the impact of cell source and processing techniques on treatment outcomes, and the development of standardized guidelines for stem cell therapy in veterinary sector need to be addressed.

## Figures and Tables

**Table 1:** Functions of pluripotent genes used to develop iPSCs.

Sl. No.	Genes	Role/Function	References
1	OCT4 (Octamer-binding transcription factor 4)	Activating and repressing the REX1 (reduced expression 1) gene Forms a regulatory complex with SOX2 and REX1 Precise level of OCT4 is critical for regulating pluripotency	[73]
2	SOX2 (SRY-box transcription factor 2)	Interacts with OCT4 and REX1 to form a complex that binds to DNA Activates the transcription of pluripotency-related genes	[73,74,75]
3	c-MYC (Cellular myelocytomatosis oncogene)	A proto gene which drives cell proliferation, essential for the self-renewal of stem cells Triggers global histone acetylation, which enables the binding of OCT4 and SOX2 to their target genes	[76,77]
4	KLF4 (Kruppel-like factor 4)	An oncogene essential for the long-term preservation of the embryonic stem cell phenotype Downregulates the expression of the tumor suppression protein p53 Activates transcription by interacting with histone acetyltransferases Suppresses cell proliferation and acts reciprocally with c-MYC	[78]
5	LIN28 (Lin-28 Homolog A)	This gene has the ability to transform human cells into induced pluripotent stem cells when combined with OCT4, SOX2, and NANOG It regulates cell growth and development	[79]
6	Nanog	Maintains the density of the pluripotent inner cell mass during embryonic development Maintains the pluripotent epiblast Prevents differentiation to primitive endoderm	[80,81]
7	REX1 (Reduced expression 1)	By interacting with the promoter regions of cyclin B1/B2, it controls their transcriptional activity Induced DRP1 phosphorylation	[82]

**Table 2:** Role of signal molecules secreted by stem cells in response to tissue damage.

SL. No.	Signaling Molecules	Role	References
1	bFGF (Basic fibroblast growth factor)	bFGF is a multifunctional growth factor that controls the proliferation and differentiation of stem cells, also promoting the preservation of their stemness, making this an essential factor of regenerative medicine and stem cell biology.	[108]
2	TGF-β1 (Transforming growth factor β1)	TGF-β1 is pivotal in regulating stem cell differentiation, influencing both the maintenance of pluripotency and the commitment to specific lineages through its complex signaling pathways and stem cell niche interactions.	[109]
3	Activin-A	A key regulator of stem cell development, Activin-A affects the ratio of pluripotency to lineage commitment in a context-dependent way. Understanding its signaling pathways is crucial for developing stem-cell-based therapies and regenerative medicine applications.	[110]
4	BMP-4 (Bone morphogenic protein 4)	BMP-4 is a pivotal factor in stem cell differentiation, influencing lineage commitment across various stem cell types and contributing to processes such as hematopoiesis and neuronal differentiation. Its versatile role underscores its importance in developmental biology and regenerative medicine.	[111]
5	HGF (Hepatocyte growth factor)	HGF is a crucial factor in the differentiation of stem cells, influencing their maintenance, lineage commitment, and regenerative capabilities. Its multifaceted roles make it a valuable target for therapeutic strategies in various diseases, particularly those involving tissue damage and regeneration.	[112]
6	EGF (Epidermal growth factor)	EGF is a flexible growth factor that affects the ability of different stem cell types to maintain their stemness and induce lineage commitment. Its specific effects depend on the cellular context and the interplay with other signaling pathways. Understanding EGF’s role in stem cell differentiation is crucial for developing targeted therapies and regenerative medicine.	[113]
7	βNGF (β nerve growth factor)	βNGF plays a critical role in stem cell differentiation, especially in neuronal and osteogenic lineages. Its significance in stem cell biology and therapeutic uses in regenerative medicine are highlighted by its capacity to control proliferation, survival, and lineage commitment.	[114]
8	Retinoic acid	Retinoic acid is a crucial regulator of stem cell differentiation, with the ability to both induce lineage commitment and sustain pluripotency depending on the cellular context. Understanding its complex signaling mechanisms is important for directing stem cell fate in regenerative medicine applications.	[115]
9	VEGF (Vascular Endothelial Growth factor)	Involved in angiogenesis, this factor is upregulated in spinal cord injuries after the administration of MSCs. In bone-marrow-derived mesenchymal stem cells, it helps in controlling the balance between osteoblast and adipocyte differentiation.	[116,117]
10	Interleukin-6	This has both anti-inflammatory and pro-inflammatory properties. It contributes to the growth of T helper cells and encourages hematopoietic stem cells to differentiate into different types of blood cells.	[118,119]
11	Interleukin-1 beta	This is involved in the therapeutic effects of MSC-associated cardiac repair. It also plays a role in the production of GDNF by Sertoli cells. It enhances cell migration and activates protein kinase cascades.	[120,121]

## Data Availability

No new data were created or analyzed in this study. Data sharing is not applicable to this article.
